# Oxygen Radical Absorbance Capacity of Different Varieties of Strawberry and the Antioxidant Stability in Storage

**DOI:** 10.3390/molecules18021528

**Published:** 2013-01-25

**Authors:** Chunyang Li, Wu-Yang Huang, Xing-Na Wang, Wen-Xu Liu

**Affiliations:** 1 Department of Functional Food and Bio-active compounds, Institute of Farm Product Processing, Jiangsu Academy of Agricultural Sciences, Nanjing 210014, Jiangsu, China; E-Mails: lichunyang@jaas.ac.cn (C.L.); xingnawang.nn@163.com (X.-N.W.); liuwenxu52@yahoo.com.cn (W.-X.L.); 2 College of Food Science and Technology, Nanjing Agricultural University, Nanjing 210095, Jiangsu, China

**Keywords:** oxygen radical absorbance capacity (ORAC), strawberry, different stages, different storage temperature, storage time

## Abstract

Total antioxidant capacity of different varieties of strawberry (Ningfeng, Ningyu, Zijin 4, Toyonoka, Benihope, Sweet Charlie) in different developmental stages (including green unripe stages, half red stages, and red ripe stages) was investigated by oxygen radical absorbance capacity (ORAC) assay. In addition, effects of the antioxidant properties of strawberry stored at 4 °C or −18 °C for a period of five months were studied. The results showed that antioxidant capacity of strawberry changed based on tested part, developmental stage, variety, and time of collection. Calyces had significantly higher ORAC values compared with fruits. Strawberry fruits had higher ORAC values during the green unripe stages than the half red stages and red ripe stages. Strawberries got higher ORAC values during short-time storage, and then decreased during long-time storage. Samples stored at −18 °C exhibited higher antioxidant capacity than those stored at 4 °C, while vacuum treatment could further increase ORAC values. The results indicated the potential market role of strawberries as a functional food and could provide great value in preventing oxidation reaction in food processing and storage for the dietary industry.

## 1. Introduction

Fruits play an important role in human nutrition and health because of their nutritional properties and bioactive principles [1]. Many epidemiological studies show that consumption of fruits is able to reduce the risk for some major human chronic diseases [2]. Strawberry is one of “super fruits”, since they can form a significant component of the diet and are a potential resource of functional foods and nutraceuticals due to their antioxidant capacity and nutritional quality [3,4].

Strawberry (*Fragaria x ananassa* Duch.) fruits are consumed in large quantities, either fresh or in prepared foods such as preserves, fruit juice, pies, ice creams, and milk shakes. Strawberries are an excellent source of vitamin C, and are also rich in bioactive phenolic compounds including flavonoids and phenolic acids [4,5,6]. These bioactive phytochemicals contribute the major health-promoting function [7]. Strawberries have shown a remarkably high scavenging activity toward chemically generated radicals, thus making them effective in inhibiting oxidation of human low-density lipoproteins [8]. Antioxidant activity of strawberries could contribute to the prevention of cancer, cardiovascular and other chronic diseases [9].

There are many methods to determine antioxidant activity, such as DPPH assay, ABTS assay, FRAP assay, DMPD assay, ORAC assay, and ESR spin trap method [10]. The DPPH assay is applicable to evaluate antioxidants in lipophilic systems [11]. The FRAP assay and reducing power assay are mainly used in aqueous systems [12]. The ABTS and ORAC assay are reliable tests to assess the total antioxidant capacity (both lipophilic and hydrophilic antioxidant capacity) [10]. Additionally, the ORAC assay is perhaps one of the most suitable methods to evaluate the *in vitro* antioxidant capacity of food products because it utilizes a biological relevant radical source and is the only method that combines both inhibition time and degree of inhibition into a single quantity [13]. In order to explore genotypic difference on antioxidant capacity, six different varieties of strawberry (Ningfeng, Ningyu, Zijin 4, Toyonoka, Benihope, Sweet Charlie) were investigated by ORAC assay. In addition, the ORAC values of Benihope fruits with different storage treatments were determined for analyzing the antioxidant stability.

## 2. Results and Discussion

### 2.1. Oxygen radical Absorbance Capacity (ORAC) of Different Varieties of Strawberries in Different Developmental Stages

The calyces, fruits of red ripe stage, half red stage, and green unripe stage from the six different varieties of strawberries collected from December 2010 to April 2011 were analyzed for their radical scavenging activity by the ORAC assay. Detail ORAC values of different strawberry samples are listed in Table 1.

**Table 1:** The ORAC values of six varieties of strawberries (fruits with different developmental stages and calyces) collected in different months.

		ORAC value (mmol Trolox/100 g fresh weight)				
Variety name	Used part	December	January	February	March	April
Toyonoka	fruit of green unripe stage	1.401 ± 0.112 ^b^	1.245 ± 0.051 ^a^	3.008 ± 0.232 ^c^	1.579 ± 0.013 ^ab^	1.308 ± 0.083 ^ab^
	fruit of half red stage	1.309 ± 0.079 ^b^	1.234 ± 0.074 ^a^	1.729 ± 0.027 ^b^	1.347 ± 0.139 ^ab^	1.027 ± 0.045 ^a^
	fruit of red ripe stage	1.257 ± 0.128 ^b^	1.433 ± 0.033 ^a^	1.916 ± 0.064 ^b^	0.795 ± 0.067 ^a^	0.909 ± 0.019 ^a^
	calyx	4.959 ± 0.240 ^g^	8.022 ± 0.239 ^c^	10.89 ± 0.542 ^e^	12.68 ± 0.771 ^e^	8.248 ± 0.242 ^g^
Benihope	fruit of green unripe stage	2.780 ± 0.081 ^e^	2.118 ± 0.199 ^a^	2.700 ± 0.057 ^c^	3.101 ± 0.279 ^c^	2.913 ± 0.284 ^c^
	fruit of half red stage	1.176 ± 0.170 ^b^	1.187 ± 0.150 ^a^	2.661 ± 0.335 ^c^	1.930 ± 0.097 ^b^	3.116 ± 0.215 ^c^
	fruit of red ripe stage	0.839 ± 0.087 ^a^	0.937 ± 0.096 ^a^	1.543 ± 0.121 ^ab^	1.169 ± 0.032 ^ab^	2.343 ± 0.192 ^bc^
	calyx	11.34 ± 0.310 ^h^	15.37 ± 1.339 ^e^	28.20 ± 1.101 ^i^	17.87 ± 0.942 ^h^	21.61 ± 2.091 ^i^
Ningfeng	fruit of green unripe stage	1.565 ± 0.057 ^b^	1.229 ± 0.128 ^a^	2.370 ± 0.336 ^bc^	1.784 ± 0.138 ^b^	4.654 ± 0.452 ^d^
	fruit of half red stage	n.d.	n.d.	1.722 ± 0.139 ^b^	0.902 ± 0.055 ^a^	3.197 ± 0.119 ^c^
	fruit of red ripe stage	n.d.	n.d.	2.069 ± 0.108 ^bc^	1.186 ± 0.147 ^ab^	2.685 ± 0.157 ^c^
	calyx	4.066 ± 0.141 ^f^	11.67 ± 0.727 ^d^	15.46 ± 1.120 ^g^	15.59 ± 1.105 ^g^	16.68 ± 0.661 ^h^
Ningyu	fruit of green unripe stage	1.738 ± 0.114 ^b^	3.392 ± 0.395 ^b^	2.706 ± 0.175 ^c^	3.623 ± 0.159 ^c^	3.288 ± 0.037 ^c^
	fruit of half red stage	1.615 ± 0.159 ^b^	2.160 ± 0.184 ^ab^	2.049 ± 0.104 ^bc^	1.270 ± 0.130 ^ab^	2.282 ± 0.092 ^bc^
	fruit of red ripe stage	1.802 ± 0.049 ^c^	1.404 ± 0.104 ^a^	1.706 ± 0.069 ^b^	1.219 ± 0.069 ^ab^	2.830 ± 0.063 ^c^
	calyx	3.420 ± 0.296 ^f^	15.40 ± 2.095 ^e^	22.64 ± 0.804 ^h^	18.78 ± 1.623 ^i^	5.592 ± 0.431 ^e^
Sweet Charlie	fruit of green unripe stage	2.321 ± 0.081 ^d^	2.451 ± 0.199 ^a^	1.608 ± 0.109 ^b^	3.437 ± 0.078 ^c^	1.815 ± 0.125 ^b^
	fruit of half red stage	1.629 ± 0.142 ^b^	1.738 ± 0.227 ^a^	1.458 ± 0.122 ^b^	1.377 ± 0.097 ^ab^	2.128 ± 0.099 ^bc^
	fruit of red ripe stage	1.303 ± 0.149 ^b^	1.408 ± 0.089 ^a^	1.286 ± 0.173 ^ab^	1.892 ± 0.175 ^b^	1.357 ± 0.131 ^ab^
	calyx	5.242 ± 0.311 ^g^	11.78 ± 1.594 ^d^	7.238 ± 0.195 ^d^	11.29 ± 0.919 ^d^	6.697 ± 0.232 ^f^
Zijin 4	fruit of green unripe stage	2.404 ± 0.098 ^d^	2.041 ± 0.192 ^a^	2.777 ± 0.080 ^c^	1.503 ± 0.039 ^ab^	2.547 ± 0.186 ^bc^
	fruit of half red stage	1.307 ± 0.070 ^b^	1.697 ± 0.837 ^a^	1.538 ± 0.101 ^ab^	0.968 ± 0.036 ^a^	2.682 ± 0.144 ^c^
	fruit of red ripe stage	1.250 ± 0.080 ^b^	1.279 ± 0.168 ^a^	0.872 ± 0.105 ^a^	0.901 ± 0.095 ^a^	1.712 ± 0.212 ^ab^
	calyx	4.980 ± 0.378 ^g^	9.759 ± 0.444 ^c^	13.57 ± 0.721 ^f^	13.91 ± 0.305 ^f^	7.352 ± 0.635 ^f^
*LSD*		0.847	1.7319	1.3179	1.4422	1.4156

n.d., not determined; *p* < 0.05 using Fisher’s *LSD* and different letters in the same column indicate significant differences among these strawberry samples.

Antioxidant capacity of strawberry changed based on tested part, developmental stage, variety, and collection time. The highest ORAC value (4.654 mmol Trolox/100 g fresh weight (FW)) of strawberry fruit was obtained from Ningfeng (NF) of green unripe stage collected in April 2011, followed by Ningyu (NY) and Sweet Charlie (SC) of green unripe stages collected in March 2011 with the ORAC values at 3.623 and 3.437 mmol Trolox/100 g FW, respectively. Besides, fruits of green unripe stages (GF) from NY collected in January and April, fruits of half red stages (HF) from NF and Benihope (BE) collected in April, Benihope’s GF collected in March, and Toyonoka’s GF collected in February exhibited high antioxidant capacity with ORAC values over 3.0 mmol Trolox/100 g FW. The lowest ORAC value of strawberry fruit was obtained from Toyonoka (TO) of red ripe stage collected in March 2011 (0.795 mmol Trolox/100 g FW). Most weak antioxidant capacities with ORAC values below 1.0 mmol Trolox/100 g FW corresponded to fruits of red ripe stages (RF). Few strawberries of HF showed weak antioxidant capacity, such as NF and ZJ collected in March with the ORAC values at 0.902 and 0.968 mmol Trolox/100 g FW, respectively. All the calyx samples showed higher ORAC values than fruit samples, except NY and NF collected in December 2010 (3.420 and 4.066 mmol Trolox/100 g FW, respectively). Strongest antioxidant capacity of strawberry calyx was obtained from BE collected in February with the ORAC value at 28.20 mmol Trolox/100 g FW.

Different varieties of strawberry possessed different antioxidant capacity. Ningfeng and Ningyu cultivars from Horticulture Research Institute, Jiangsu Academy of Agricultural Sciences had better antioxidant capacity than others. Mean ORAC values of fruits in the three different stages from December 2010 to April 2011 showed Benihope, Ningfeng, and Ningyu significantly higher than Toyonoka (*p* < 0.001). The mean values of TO, BE, NF, NY, SC, and ZJ fruits were 1.433, 2.034, 2.124, 2.206, 1.814, and 1.699 mmol Trolox/100 g FW, respectively. While their calyces had mean values at 8.627, 19.14, 12.81, 13.30, 8.451, and 9.913 mmol Trolox/100 g FW, respectively. Significant differences existed in calyx antioxidant capacity between Benihope and the other five varieties (*p* < 0.05). Although fruits of green unripe stages did not always possess better antioxidant capacity than fruits of half red and red ripe stages, mean ORAC values of fruits in the six different varieties from December 2010 to April 2011 showed significant difference among these three developmental stages (compared to RF: HF, *p* < 0.05; GF, *p* < 0.001). In general, GF showed better antioxidant capacity than HF, and HF were better than RF. Their values were 2.380, 1.730, and 1.475 mmol Trolox/100 g FW for GF, HF, and RF, respectively.

ORAC values of strawberries collected from different time changed greatly but not so regularly. Fruits of different varieties seemed to show low antioxidant capacity in December 2010 and high antioxidant capacity in February 2011, with the total mean ORAC values of three developmental stages from six varieties at 1.602 and 1.995 mmol Trolox/100 g FW, respectively. In addition, the difference among fruits of the six varieties collected in April 2011 was great significant (*p* < 0.001) with mean ORAC values from 1.081 to 3.512 mmol Trolox/100 g FW (except BE and NY, 2.791 and 2.800, respectively), and the total mean value (2.380 mmol Trolox/100 g FW) was also high. The ORAC values of calyces were relatively high and the differences among varieties were significant from January to March 2011, especially February (28.20, 22.64, 15.46, 13.57, 10.89, and 7.238 mmol trolox/100 g FW for BE, NY, NF, ZJ, TO, and SC, respectively, *p* < 0.001). Calyces of BE possessed significantly stronger antioxidant capacity than other five varieties during all the months from December 2010 to April 2011, except that NY showed similar high ORAC values in January and March 2011. Calyces’ ORAC values of four varieties (TO, NY, SC, and ZJ) in April 2011 and five varieties (except BE) were relative lower than others. Two-way ANOVA with post tests showed significant difference among varieties and collected months for both fruits and calyces (source of variation: interaction, both *p* < 0.001), which indicated that varieties and collected time were important effect on ORAC values of strawberry. ORAC value changes of fruits in different stages collected from December 2010 to April 2011 also showed that GF’s antioxidant capacity (mean ORAC of six varieties all over 2.0 mmol Trolox/100 g FW) significantly better than HF’s and RF’s (*p* < 0.01). However, two-way ANOVA not showed significant difference among developmental stages and collected months (*p* > 0.05).

Many factors could potentially account for the differences in antioxidant capacity, such as species, varieties, material origins, cultivation conditions, developmental stages, and so on. In general, genotype and origin proved to have a greater effect than the cultivation techniques on parameter levels [14]. Panico *et al.* [15] reported the influence of soil on fruit quality and the difference on phytochemicals and antioxidant activity of two genotypes of strawberry fruit, one represented by the cultivated variety (cv) “Tudla” and the other one, “Maletto”, by a type selected in the mountain region of Etna (Italy). Carbone *et al.* [16] also reported influence of internal (genetic and developmental) and external (environmental) factors on levels of flavonoid gene transcripts, enzyme activity and metabolites of six cultivated strawberry genotypes grown at two Italian locations. They found that flavonoid metabolite profiles were strongly affected by genotype during strawberry fruit development, and environmental factors also the major impact of flavonoid metabolism and developmental processes. In our study, two of three new strawberry breeding varieties from Horticulture Research Institute, Jiangsu Academy of Agricultural Sciences, Ningfeng and Ningyu cultivars had better antioxidant capacity than others. Fruits of green unripe stages possessed better antioxidant capacity than fruits of half red and red ripe stages, which was consistent with previous reports. Wang and Lin [17] also found strawberries and blackberries had the highest ORAC values during the green stages, and leaves showed better antioxidant capacity than fruits. Similar to leaves, calyces in the present study were found to have higher ORAC values compared with fruits.

### 2.2. Primary Identification of Phenolic Constituents

In the present study, then mean value of total phenolic content from methanolic extracts of TO, BE, NF, NY, SC, and ZJ fruits were 1.97, 2.59, 2.89, 3.77, 2.46, and 2.28 mg gallic acid/g FW, respectively. While their calyces had mean values at 10.62, 19.63, 15.64, 16.23, 11.23, and 14.83 mg gallic acid/g FW, respectively (Table 2). A highly positive linear correlation between total phenolic content (*y*) and total antioxidant capacity (*x*) was established for the strawberry fruits and calyces (*y* = 1.1017*x* + 1.0078, *R^2^* = 0.964). The linear correlation between total phenolic content and ORAC activity indicated phytochemicals were responsible for the antioxidant capacity [17]. These phenolic compounds (including phenolic acids, flavonoids, and anthocyanidins) in the sampled strawberry significantly contributed to their total antioxidant capacity, which was consistent with previous reports [18,19].

**Table 2:** The total phenolic compounds of fruits and calyces from six varieties of strawberries.

Sample	TPC (mg gallic acid/g)						
	TO	BE	NF	NY	SC	ZJ	Mean
Fruits	1.97 ± 0.19	2.59 ± 0.14	2.89 ± 0.14	3.77 ± 0.20	2.46 ± 0.13	2.28 ± 0.06	2.66 ± 0.60
Calyces ***	10.62 ± 0.31	19.63 ± 0.34	15.64 ± 0.12	16.23 ± 0.14	11.23 ± 0.17	14.83 ± 0.16	14.7 ± 3.16

Asterisks (***) denote the values for fruits and calyces were significantly different (*p* < 0.001).

**Figure 1:**
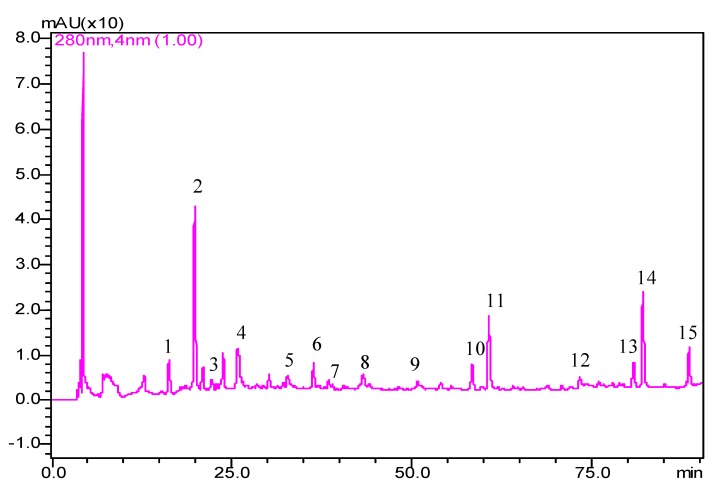
HPLC profiles of Toyonoka strawberries. Peaks: 1: gallic acid; 2: gallocatechin; 3: protocatechuic acid; 4: epigallocatechin; 5: catechin; 6: p-hydroxybenzoic acids; 7: caffeic acid; 8: malvidin-3-glucoside; 9: p-coumaric acid; 10: catechingallate; 11: cyanidin; 12: ellagic acid; 13: quercetrin (quercetin-3-rhamnoside); 14: cinnamic acid; 15: luteolin.

We analyzed major phenolic compounds of strawberry using RP-HPLC with DAD by comparison with authentic phenolic standards and related literature data [18,19]. The preliminary results showed that major types of phenolic compounds in strawberry included phenolic acids, flavonoids (including flavones, flavonols, flavanols, as well as anthocyanidins) and their derivatives. For example, HPLC chromatographs of major phenolic compounds in the Toyonoka samples are shown in Figure 1 (other data are not shown). Fifteen detail phenolic compounds were eluted with *t*_R_ from 15.236 min to 88.134 min as the following order: gallic acid, gallocatechin, protocatechuic acid, epigallocatechin, catechin, *p*-hydroxybenzoic acids, caffeic acid, malvidin-3-glucoside, *p*-coumaric acid, catechin gallate, cyaniding, ellagic acid, quercetrin (quercetin-3-rhamnoside), cinnamic acid, luteolin. Most detected phenolic compounds in this study have previously been reported in strawberry of North and South America [19,20].

Different phenolic compounds normally possess specific chromatographic behavior (retention time, *t*_R_) and UV-visible spectral characteristic (λ_max_ and spectral shapes). Because of the diversity and complexity of the natural mixtures of phenolic compounds, it is rather difficult to characterize every compound and elucidate its structure. Therefore, only a preliminary identification of major phenolic compositions was carried out in this study. The dominant phenolic compounds identified in this study were phenolic acids, catechins (flavanols), and other flavonoids. Flavanols contain a variety of phenolic hydroxyl groups and exhibit the strongest antioxidant capacity and free scavenging activity among around a hundred of phenolic compounds [18]. Phenolic acids also have high antioxidant capacity, which decreased in the order protocatechuic acid > caffeic acid > *p*-hydroxybenzoic acid > ferulic acid > vanillic acid > *p*-coumaric acid [19]. These major phenolic compounds are likely the most significant contributors to the total antioxidant capacity of the strawberry samples tested in this study.

### 2.3. Effect of Storage on the Antioxidant Properties

**Figure 2:**
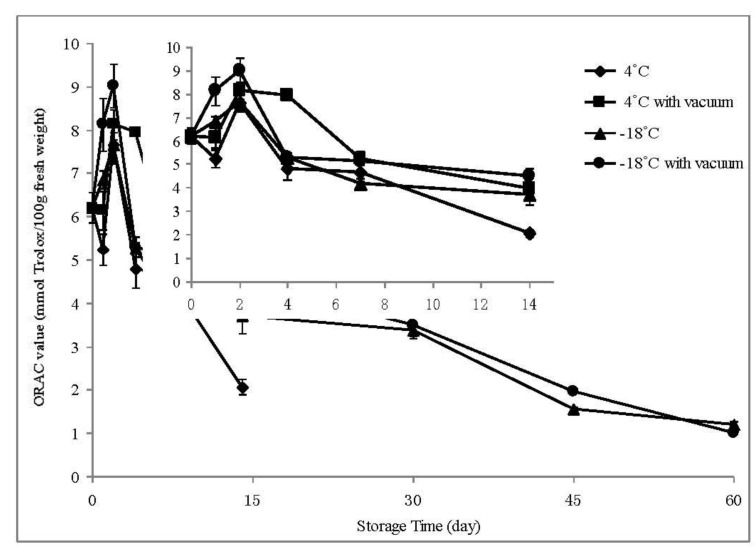
The temporal trend on oxygen radical absorbance capacity of Benihope strawberry samples stored at 4 °C and −18 °C with or without vacuum. Bars represent mean values ± SD, n = 3 separate experiments. The upper right is enlargement graph within 14 day.

Benihope with high ORAC values for both fruits and calyces was chosen to investigate the effect of storage on the antioxidant properties. Fruits of BE were stored at 4 °C or −18 °C with or without vacuum treatment. Samples at room temperature could only be stored for one day because of rot, so after collection ORAC values were detected within eight hours as data of Day 0. Fruits of BE stored at 4 °C were investigated within 14 days since parts of them also rotted. The temporal trend on oxygen radical absorbance capacity is showed in Figure 2.

In general, the antioxidant capacity of Benihope strawberry samples decreased over time gradually except the first two days. Samples at room temperature was detected on Day 0 had ORAC value of 6.204 mmol Trolox/100 g FW. All the BE samples got the highest ORAC values on the second day. The mean ORAC values of BE samples stored at 4 °C without and with vacuum treatment, −18 °C without and with vacuum treatment were 7.644 (23.22% increase), 8.122 (30.93% increase), 7.646 (23.25% increase) and 9.015 mmol Trolox/100 g FW (45.32% increase) on Day 2, respectively. From Day 4, ORAC values of BE samples began lower than that of Day 0, except that BE stored at 4 °C with vacuum treatment showed a little higher ORAC value (7.944 mmol Trolox/100 g FW). On Day 14, the ORAC value was decreased to 2.060 mmol Trolox/100 g FW (66.79% decrease) as stored at 4 °C, 3.955 mmol Trolox/100 g FW (36.25% decrease) as stored at 4 °C with vacuum treatment, 3.698 mmol Trolox/100 g FW (40.39% decrease) as stored at −18 °C, and to 4.511 mmol Trolox/100 g FW (27.29% decrease) as stored at −18 °C with vacuum treatment, respectively. After Day 30, the antioxidant capacity of BE samples was decreased greatly (>50% decrease). The ORAC value was only 1.206 and 0.999 mmol Trolox/100 g FW (80.55% and 83.89% decrease) on Day 60 for BE stored at −18 °C without and with vacuum treatment, respectively.

Most samples stored at −18 °C possessed stronger antioxidant capacity and could stored more time than those stored at 4 °C. Except BE stored for 4 days (with vacuum treatment) and 7 days (with or without vacuum treatment), all other samples stored at −18 °C showed higher ORAC values than those stored at 4 °C. Especially on Day 14, ORAC value of BE stored at −18 °C was 79.52% higher than that stored at 4 °C. In addition, vacuum treatment could increase ORAC value of BE samples to some extent. BE samples with vacuum treatment both stored at 4 °C and −18 °C showed higher (0.46%~91.96% increase) ORAC values than those without vacuum treatment except Day 60 (−17.19%). However, one-way ANOVA with Tukey’s *post hoc* test showed no significant difference among these four groups.

Antioxidant activity of food could be influenced by storage time and temperature. In the present study, the highest ORAC values were observed on the second day, and then the antioxidant capacity of Benihope strawberry samples decreased over time gradually. There was also an increase in the antioxidant capacity of fresh fruits and vegetables during short-time storage [21,22], however, long-time storage led to loss of antioxidant capacity due to phenolics degradation [23]. Among the factors that can influence the levels of antioxidant activity and total phenolic compounds, temperature was found to be a dominant factor [24]. Storing under refrigeration could better preserve antioxidant capacity since it had higher ORAC values than that in room temperature [25]. In this study, strawberries showed relatively higher ORAC values and stability stored at −18 °C than those stored at 4 °C. In addition, vacuum treatment might be another factor to preserve antioxidant capacity of strawberry samples during the storage [26].

## 3. Experimental

### 3.1. Plant Materials

Six different varieties of strawberry, including Ningfeng (NF), Ningyu (NY), Zijin 4 (ZJ), Toyonoka (TO), Benihope (BE), and Sweet Charlie (SC), were harvested from Lishui Botanic Garden, Jiangsu Academy of Agricultural Sciences in Nanjing, China. NF, NY, ZJ were three new breeding cultivars from Horticulture Research Institute, Jiangsu Academy of Agricultural Sciences. TO from Japan was cultivated in Nanjing in 2003. BE was breeding variety hybridized by two Japanese varieties, paternal Sachinoka and maternal Akihime. SC was introduced from USA. The fresh fruits in different developmental stages were randomly picked from several plants in the greenhouse during the period of December 2010 to April 2011, which included red ripe stage (R), half red stage (H), and green unripe stage (G).

### 3.2. Chemicals and Reagents

Trolox (6-hydroxy-2,5,7,8-tetramethylchromate-2-carboxylic acid) was obtained from Acros Organics (Morris Plains, NJ, USA). Fluorescein disodium was purchased from Chemical Industry of East China Normal University (Shanghai, China), and AAPH (2,2'-azobis(2-methylpropionamide)-dihydrochloride) was obtained from J&K Chemical Ltd. (Beijing, China). All other chemicals and reagents used in this study were obtained from China and were of analytical grade.

### 3.3. Sample Preparation

The fresh fruit sample (2 g) in 80% methanol (20 mL) and the calyx sample (0.5 g) in 80% methanol (10 mL) were incubated at 28 °C for 24 h in a THZ-Q Shaking Incubator (Huamei Biochem Inc., Taicang, Jiangsu, China) at 120 rpm for extraction. The extract was filtered with a medium-speed filter paper under vacuum at room temperature, and stored at −16 °C until analysis.

### 3.4. Determination of Total Phenolic Content

Total phenolic content (TPC) was estimated using the Folin-Ciocalteu colorimetric method described by Huang *et al.* [11]. Quantification was done on the basis of the standard curve of gallic acid. Results were expressed as gallic acid equivalent (GAE), *i.e.*, mg gallic acid/g dried weight (DW).

### 3.5. Phenolic Composition by Reversed Phase-High Performance Liquid Chromatography (RP-HPLC)

RP-HPLC analysis was performed using a Shimadzu HPLC System (Shimadzu LC-10A series, Tokyo, Japan), consisting of a binary pump, and a diode-array detector (DAD) and equipped with a Shim-pack RP-C18 column (5 μm, 250 × 4.6 mm) (Shimadzu, Co.). Sample was injected into LC for analysis. Phenolic compounds in the samples were analyzed at 35 °C with the following gradient elution program (solution A, 0.1% formic acid, and solution B, 100% methanol): 0-10 min, 0%-10% B; 10–25 min, 10%–20% B; 25–35 min, 20%–23% B; 35–45 min, 23%–28% B; 45–60 min, 28%–35% B. 60–75 min, 35%–50% B; 75–80 min, 50%–55% B; 80–85 min, 55%–75% B; 85–90 min, 75% B. Flow rate was 0.8 mL/min and injection volume was 10 μL. Detection was monitored at 280 nm.

### 3.6. Radical Scavenging Activity by ORAC Assay

The fruit or calyx extract was suitably diluted and assayed by the improved oxygen radical absorbance capacity (ORAC) method using fluorescein as fluorescent probe [27]. Briefly, the reaction was carried out at 37 °C in 75 mM phosphate buffer (pH 7.4). Totally 100 μL of antioxidant (Trolox or sample at different concentrations) and 50 μL of fluorescein (0.1 µM, final concentration) were placed in the well of the black 96-well microplates. The mixture was pre-incubated for 15 min at 37 °C. AAPH solution (50 μL; 60 mM) was added rapidly using a multichannel pipette. The plate was automatically shaken before the first reading, and the fluorescence was recorded every minute for 100 minutes. LB 941 TriStar Microplate Reader (Berthold Technologies, Bad Wildbad, Germany) with 485-P excitation and 535-P emission filters was controlled by MikroWin Microplate Data Reduction 2000 (Mikrotek Laborsysteme GmbH, Overath, Germany) for fluorescence measurement. A blank using phosphate buffer instead of the antioxidant solution and six calibration solution using trolox standard (0, 2, 4, 8, 12, 16 μM) were carried out in each assay. Final ORAC values were expressed as trolox equivalent (TE), *i.e.*, mmol Trolox/100 g fresh weight (FW).

### 3.7. Effect of Storage on the Antioxidant Properties

The fresh strawberry Benihope was collected from greenhouse in Lishui Botanic Garden on May 30th. The ripe fruits were brought back to the lab immediately and all packed into PET (polyester terephthalate) plastic bags. The fruit samples randomly grouped into three sets. One set was stored at room temperature and determined ORAC value within eight hours as data of Day 0. The other two sets both included two groups, with or without vacuum treatment using Sinbo Vacuum Sealer (Yindu Packaging Machinery Ltd., Wenzhou, Zhejiang, China). One set was stored at 4 °C, and the other set was at −18 °C. The ORAC values with different storage treatments were detected on Day 1, 4, 7, 10, 14, 30, 45, and Day 60, respectively.

### 3.8. Statistical Analysis

All sample determinations were conducted in triplicate and the results were calculated as mean values ± standard deviation (SD) in this study. The fluorescence decay curves were original data from MikroWin Microplate Data Reduction 2000. Coefficients of determination (*R^2^*) were calculated using Microsoft Excel 2003. Data figures were obtained using GraphPad Prism Version 5.01 (GraphPad Software, Inc., San Diego, CA, USA). ANOVA with Tukey’s post hoc test was used to determine statistical differences of one factor, while two-way ANOVA was used to analyze differences of two factors. Differences with *p* value < 0.05 were considered significant.

## 4. Conclusions

Oxygen radical absorbance capacity assays confirmed that strawberries were a good source of natural antioxidants. Different varieties of strawberry in different developmental stages had different ORAC values. The phenolic compounds were responsible for their total antioxidant capacity. Strawberry fruits had the highest ORAC values during the green unripe stages, and calyces had higher ORAC values compared with fruits. Oxygen radical scavenging effect of strawberry during storage was associated with storage time and temperature. There was a little increase on ORAC values of strawberries during short-time storage, but a gradual decrease during long-time storage. Strawberries showed relatively higher ORAC values and stability stored at −18 °C than those stored at 4 °C. In addition, vacuum treatment seemed to be helpful to preserve antioxidant capacity of strawberry samples during the storage. Data reported in this study could be of great value for the dietary industry in enhancing antioxidant properties of functional foods and in preventing oxidation reaction in food processing and storage, which would benefit human nutrition and health.
